# Supplementary material for: Efficient Fetal-Maternal ECG Signal Separation from Two Channel Maternal Abdominal ECG via Diffusion-Based Channel Selection
Source: Front Physiol. 2017 May 16;8:277. doi: 10.3389/fphys.2017.00277 (PMC5432652; doi:10.3389/fphys.2017.00277)
Supplement: Supplementary file 1 [file DataSheet1.pdf]

SI.1 THEORETICAL BACKGROUND FOR THE CHANNEL SELECTION

We describe the background material needed for the proposed two-channel fECG algorithm. The algorithm is composed of three essential components. The first component is estimating the maternal cardiac activity in the aECG (maECG) by applying the dsSTFT [1], the beat tracking and the nonlocal median [2], and get a rough fECG from any given linear combination of the two provided aECG signals. The second component is the channel selection by applying the lag map [3, 4, 5] and diffusion map (DM) [6] for the sake of determining the best linear combination of the two channels, which lead to the optimal rough fECG. The third component is getting the fECG and fetal R peaks information from the optimal rough fECG by again the dsSTFT and the beat tracking.

SI.1.1 Linear combination

The main motivation behind the algorithm is motivated by the physiological knowledge of the ECG signal that among all linear combinations of two channels, with a high probability we could find a combination that is optimal for the fetal ECG extraction.

Before describing the linear combination idea, recall the well-know *vectocardiogram* (VCG) and its relationship with the ECG signals. It has been well known that the ECG signal, denoted as a continuous time series $E : [0, T] \to \mathbb{R}$, where $T > 0$ is the observation time, is the projection of the representative dipole current of the electrophysiological cardiac activity on a predesigned direction [7]. Denote the dipole current as a three dimensional continuous time series $d : [0, T] \to \mathbb{R}^3$. If we could record $d(t)$, it is called the VCG signal. Physiologically, for a normal subject, $d(t)$ is oscillatory with the period $\tau > 0$, which is about 1 second, in the sense that $d(t) \sim d(t + \tau)$ for all $t \in [0, T - \tau]$. Suppose t_l, $l = 1, \ldots, m$, where m is the number of cardiac cycles over the period $[0, T]$, is the timestamp corresponding to the maximal amplitude point of the l-th cardiac cycle. We call the vector

$$c = \frac{1}{m}\sum_{l=1}^{m} d(t_l) \tag{S.1}$$

the cardiac axis. For a given ECG signal, there is an associated projection direction $v \in \mathbb{R}^3$ so that E is the projection of $d(t)$ on v; that is, $E(t) = v^T d(t)$. It has been well known that depending on v, we could acquire different aspects of the cardiac information. We mention that in general, v changes according to time due to the cardiac axis deviation caused by the respiratory activity and other physical movements. To simplify the discussion, we do not take these facts into account.

Denote d_m to be the mother's VCG and d_f to be the fetus' VCG. Denote c_m to be the mother's cardiac axis and c_f to be the fetus' cardiac axis. Fix two abdominal lead placements and record two aECG signals, denoted as x_1 and x_2. Denote $v_{m,i} \in \mathbb{R}^3$ and $v_{f,i} \in \mathbb{R}^3$ to be the projection directions of the mother's VCG and fetus' VCG corresponding to x_i, where $i = 1, 2$. Obviously, we have $x_i = v_{m,i}^T d_m + v_{f,i}^T d_f$, where $i = 1, 2$, and it is possible that the fetal cardiac activity is relatively weak in both x_1 and x_2. To resolve this problem, we consider the following linear combination scheme. Take a linear combination of x_1 and x_2 by

$$\begin{aligned} x_\theta &= \theta x_1 + \sqrt{1-\theta^2} x_2 \\ &= \theta v_{m,1}^T d_m + \theta v_{f,1}^T d_f + \sqrt{1-\theta^2} v_{m,2}^T d_m + \sqrt{1-\theta^2} v_{f,2}^T d_f \\ &= [\theta v_{m,1}^T + \sqrt{1-\theta^2} v_{m,2}^T] d_m + [\theta v_{f,1}^T + \sqrt{1-\theta^2} v_{f,2}^T] d_f, \end{aligned} \tag{S.2}$$

where $\theta \in (-1, 1]$. If these two abdominal leads are placed on two different locations, we know $v_{f,1} \neq v_{f,2}$ and $v_{m,1} \neq v_{m,2}$, and hence the set

$$A := \{\theta v_{f,1} - \sqrt{1-\theta^2} v_{f,2}\}_{\theta \in (-1,1]} \tag{S.3}$$

contains all linear combinations of $v_{f,1}$ and $v_{f,2}$ if we do not distinguish $\theta v_{f,1} - \sqrt{1-\theta^2} v_{f,2}$ and $-\theta v_{f,1} + \sqrt{1-\theta^2} v_{f,2}$. Note that the set $\{(\theta, \sqrt{1-\theta^2})\}_{\theta \in (-1,1]}$ is the 1-dimensional real projective space (identifying antipodal points of the unit circle, $S^1 := \{x \in \mathbb{R}^2 | \|x\| = 1\}$, embedded in $\mathbb{R}^2$), and it topologically equivalent to the unit circle S^1, which is an one dimensional manifold. Based on the above-mentioned relationship between ECG and VCG, although the fetus could rotate inside the uterus, we know that unless the cardiac axis c_f of the fetal cardiac activity is perpendicular or almost perpendicular to both $v_{f,1}$ and $v_{f,2}$, we could find an θ so that x_θ contains a strong fetal cardiac activity. Since the chance that the fetal cardiac axis is perpendicular to the 2-dim affine subspace corresponding to the two chosen abdominal leads is low, we could thus conclude that the chance that we could obtain a good signal with strong fECG via the linear combination scheme is high.

SI.1.2 Lag map

The lag map is a well-known method widely applied to study a given time series, and its theoretical foundation has been well established in [3, 8, 9]. In brief, it allows us to reconstruct the structure underlying the time series. For a given time series $\boldsymbol{f}$ of length $N \in \mathbb{N}$, the lag map is a mapping from $\boldsymbol{f}$ to a set of L-dim points, where L is chosen by the user, via

$$\Psi_{\boldsymbol{f},L} : i \mapsto (\boldsymbol{f}(i), \ldots, \boldsymbol{f}(i+L))^T \in \mathbb{R}^{L+1}, \tag{S.4}$$

where $i = 1, \ldots, N-L$ and the superscript T means taking the transpose. The map $\Psi_{\boldsymbol{f},L}$ is called the *L-step lag map*. It has been shown in [3] that if $\boldsymbol{f}$ is an observation of a dynamical process whose trajectory is supported on a d dimensional manifold and L is large enough, then under some weak mathematical conditions, $\Psi_{\boldsymbol{f},L}$ could recover the manifold up to a diffeomorphism. Since the cardiac activity is periodic, the corresponding "underlying manifold" is a one-dimensional circle representing the cardiac dynamics that is diffeomorphic to the unit circle S^1, and the lag map of the cardiac activity time series leads to a point cloud supported on another one-dimensional simple closed curve.

The above-mentioned important property of the lag map allows up to examine the quality of the reconstructed fECG. If $\boldsymbol{f} \in \mathbb{R}^N$ is the true fECG signal, or a good estimation of the fECG signal, we obtain an one-dimensional simple closed curve by the point cloud $\mathcal{X}_{\boldsymbol{f},L} := \{\Psi_{\boldsymbol{f},L}(i)\}_{i=1}^{N-L} \in \mathbb{R}^{L+1}$. On the other hand, if the tempted fECG estimator $\boldsymbol{f} \in \mathbb{R}^N$ fails to be a good estimator of the fECG signal, the point cloud $\mathcal{X}_{\boldsymbol{f},L}$ might be away from any one-dimensional simple closed curve. Another important fact is that when $\boldsymbol{f}$ is the fECG signal, the point cloud $\mathcal{X}_{\boldsymbol{f},L}$ is in general non-uniformly sampled from the one-dimensional circle. This fact comes from the diffeomorphic relationship between the reconstructed simple closed curve and the underlying simple closed curve via the lag map.

SI.1.3 Graph Laplacian and diffusion map

To take this important fact into account to examine the quality of the reconstructed fECG via the L-step lag map, we apply the graph Laplacian (GL), which is the building block of several dimension reduction algorithms, like the diffusion map (DM) [6, 10]. For a general introduction of GL and DM, we refer readers to [6, 11, 10]. Here we only provide the necessary steps for our purpose. Fix the given embedded point

cloud $\mathcal{X}_{\boldsymbol{f},L}$. Build a complete affinity graph $G = (V, E, \omega)$ with vertices $V = \mathcal{X}_{\boldsymbol{f},L}$ by viewing any pairs of points in $\mathcal{X}_{\boldsymbol{f},L}$ as edges; that is $E = \{(\Psi_{\boldsymbol{f},L}(i), \Psi_{\boldsymbol{f},L}(j)) | \, i \neq j\}$. The affinity function $\omega : E \to \mathbb{R}^+$ is then defined by

$$\omega(\Psi_{\boldsymbol{f},L}(i), \Psi_{\boldsymbol{f},L}(j)) = \exp\Big\{ - \frac{\|\Psi_{\boldsymbol{f},L}(i) - \Psi_{\boldsymbol{f},L}(j)\|^2}{\epsilon}\Big\}, \tag{S.5}$$

for $i, j = 1, \ldots, N - L$, $i \neq j$, and $\epsilon > 0$ is the kernel bandwidth chosen by the user. Here, the affinity between $\Psi_{\boldsymbol{f},L}(i)$ and $\Psi_{\boldsymbol{f},L}(j)$ is reversely proportional to the distance between $\Psi_{\boldsymbol{f},L}(i)$ and $\Psi_{\boldsymbol{f},L}(j)$. Note that while we could choose a more general kernel, here we focus on the Gaussian kernel to simplify the discussion. We mention that in practice the Gaussian kernel performs well and the dependence on the chosen kernel is marginal. In general, the point cloud might not be uniformly sampled from the geometric object we have interest, and the nonuniform sampling effect might generate a negative impact on the upcoming analysis. To resolve this issue, the α-normalization technique is introduced in [6]. Take $0 \leq \alpha \leq 1$, we could define an α-normalized affinity function defined on E, denoted as $\omega^{(\alpha)}$, by

$$\omega^{(\alpha)}(\Psi_{\boldsymbol{f},L}(i), \Psi_{\boldsymbol{f},L}(j)) = \frac{\omega(\Psi_{\boldsymbol{f},L}(i), \Psi_{\boldsymbol{f},L}(j))}{d_i^\alpha d_j^\alpha}, \tag{S.6}$$

where d is the degree function defined on the vertex set as

$$d_i = \sum_{j=1}^{N-L} \omega^{(\alpha)}(\Psi_{\boldsymbol{f},L}(i), \Psi_{\boldsymbol{f},L}(j)), \tag{S.7}$$

for $i = 1, \ldots, N - L$. As is shown in [6, 10], when $\alpha = 1$, this α-normalized affinity could effectively alleviate the impacts introduced by the nonuniform sampling. In our fECG application, as discussed above, $\mathcal{X}_{\boldsymbol{f},L}$ is in general non-uniformly sampled from the one-dimensional simple closed curve, so we apply this α-normalization technique.

We are now ready to define the GL. Define an α-normalized affinity matrix $W^{(\alpha)} \in \mathbb{R}^{(N-L)\times(N-L)}$ by

$$W_{ij}^{(\alpha)} := \omega^{(\alpha)}(\Psi_{\boldsymbol{f},L}(i), \Psi_{\boldsymbol{f},L}(j)), \tag{S.8}$$

for $i, j = 1, \ldots, N - L$, define a diagonal α-normalized degree matrix $D^{(\alpha)} \in \mathbb{R}^{(N-L)\times(N-L)}$ by

$$D_{ii}^{(\alpha)} = \sum_{j=1}^{N-L} W_{ij}, \tag{S.9}$$

for $i = 1, \ldots, N - L$. and the *α-normalized graph Laplacian* is then defined by

$$L^{(\alpha)} := I - {D^{(\alpha)}}^{-1} W^{(\alpha)}. \tag{S.10}$$

Since L is similar to the symmetric matrix $I - {D^{(\alpha)}}^{-1/2} W^{(\alpha)} {D^{(\alpha)}}^{-1/2}$, it has a complete set of right eigenvectors $\phi_1, \ldots, \phi_{N-L}$ with corresponding eigenvalues $0 = \lambda_1 < \lambda_2 \leq \cdots \leq \lambda_{N-L} \leq 1$. Note that $\phi_1 = (1, 1 \ldots, 1)^T$ since ${D^{(\alpha)}}^{-1} W^{(\alpha)}$ is a transition matrix defined on the graph G. It has been shown in [6, 10] that if $\mathcal{X}$ is sampled from a low dimensional Riemannian manifold, when $\alpha = 1$ and $N \to \infty$, asymptotically the eigenvectors ϕ_i converges pointwisely and spectrally to the i-th eigenfunction of the Laplace-Beltrami operator of the Riemannian manifold. In general, this allows us to reconstruct

the manifold by applying the diffusion geometry and the spectral embedding theory, which is commonly known as the DM algorithm [6]. The robustness of the GL and DM has been studied in [12, 11].

In our problem, due to the periodic oscillation intrinsic to the fECG we have interest, the α-normalized graph Laplacian associated with $\mathcal{X}_{f,L}$ gives us the Laplace-Beltrami operator over a simple closed curve. It follows that asymptotically, the first two non-trivial eigenvectors are the sine and cosine functions. We could thus take this fact into account and design the signal quality index for the channel selection purpose.

REFERENCES

1 .Lin CY, Li S, Wu HT. Wave-shape function analysis–when cepstrum meets time-frequency analysis. *Journal of Fourier Analysis and Applications* **accepted for publication** (2016).

2 .Su L, Wu HT. Extract fetal ECG from single-lead abdominal ECG by de-shape short time Fourier transform and nonlocal median. *Frontiers in Applied Mathematics and Statistics* **3** (2017) 2. doi:10.3389/fams.2017.00002.

3 .Takens F. Detecting strange attractors in turbulence. Rand D, Young LS, editors, *Dynamical Systems and Turbulence* (Springer Berlin Heidelberg), *Lecture Notes in Mathematics*, vol. 898 (1981), 366–381.

4 .Richter M, Schreiber T, Kaplan D. Fetal ECG extraction with nonlinear state-space projections. *IEEE Trans. Biomed. Eng.* **45** (1998) 133–137.

5 .Kotas M, Jezewski J, Matonia A, Kupka T. Towards noise immune detection of fetal QRS complexes. *Comput Methods Programs Biomed* **97** (2010) 241–256.

6 .Coifman RR, Lafon S. Diffusion maps. *Appl. Comput. Harmon. Anal.* **21** (2006) 5–30.

7 .Keener J. *Mathematical Physiology* (Springer) (1998).

8 .Stark J, Broomhead D, Davies M, Huke J. Takens embedding theorems for forced and stochastic systems. *Nonlinear Analysis: Theory, Methods & Applications* **30** (1997) 5303–5314.

9 .Stark J, Broomhead D, Davies M, Huke J. Delay Embeddings for Forced Systems. II. Stochastic Forcing. *Journal of Nonlinear Science* **13** (2003) 519–577.

10 .Singer A, Wu HT. Spectral convergence of the connection laplacian from random samples. *Information and Inference: A Journal of the IMA* **in press, doi: 10.1093/imaiai/iaw016** (2016).

11 .El Karoui N, Wu HT. Connection graph Laplacian methods can be made robust to noise. *Ann. Stat.* **44** (2016) 346–372.

12 .El Karoui N. On information plus noise kernel random matrices. *The Annals of Statistics* **38** (2010) 3191–3216.
